# Abstracts from Foreign Journals

**Published:** 1901-06

**Authors:** M. P. Ravenel, S. J. J. Harger

**Affiliations:** Philadelphia, Pa.; Philadelphia, Pa.


### ABSTRACTS FROM FOREIGN JOURNALS.

### FRENCH.

UNDER THE DIRECTION OF M. P. RAVENAL, M.D., AND S. J. J. HARGER, V.M.D., OF PHILADELPHIA, PA.

HEREDITARY MONSTROSITY. Several Welsh ewes were served by a ram of the same breed. The latter when killed was found to have but one kidney. Afterward several of his offspring which were killed showed the same anomaly.—*L'Echo Vétérinaire.*

CLINICAL STUDY OF THE SYMPTOMS OF SHOULDER LAMENESS. Hunting has made a clinical study of the symptoms of shoulder lameness as compared with lameness in other regions where these same symptoms may exist.

1. *Circumduction.* This is the most constant symptom of shoulder lameness, but it may also be seen in diseases of the knee and the foot, where flexion of these joints is painful.

2. *Dragging the Toe upon the Ground.* This indicates inability to raise the leg, and is perhaps the most characteristic symptom of shoulder lameness, although it may also accompany lesions of the antibrachial region.

3. *Length of the Step.* The step is always short, but this is so in many other lamenesses of the anterior member.

4. *Abduction and Adduction.* Resting the foot outside of the median line is frequently seen in lameness of the foot and also in lesions of the pectoral muscles. Resting the foot within the median line is rare. In one case, the attitude being normal at rest, the foot was adducted during walking; trotting was impossible, and the necropsy revealed a fracture of the first dorsal vertebra.

5. *Passive Movements.* Manually moving the leg forward› backward, and outward only obscures the diagnosis, especially in nervous horses.

6. *Palpation.* Pressure with the fingers or the hand is most deceptive, because all horses will " give " to pressure, and the two shoulders must be compared. This is especially so if a blister has been previously applied.

7. *Position of the Member at Rest.* This gives little information as to the seat of the lameness. Generally resting the foot in front of or behind the median line signifies nothing as to the location, because these attitudes are observed in other lamenesses.

The practitioner may be firmly and justly convinced that the horse is lame in the shoulder, but he is incapable of naming a characteristic symptom. The horse may be rheumatic in the shoulder, but how could one locate it or give a convincing reason for or against the diagnosis of another practitioner? The action, palpation, and passive movements of the leg confirm or contradict the diagnosis at will. The author confesses his inability to diagnose rheumatism of the shoulder; it can be surmised but not demonstrated. He has seen obscure lameness follow idiopathic or symptomatic œdema, and at the necropsy found an alteration of the subscapular lymphatic ganglia. The differential diagnosis in such cases is difficult.

CONCLUSIONS. When no cause of lameness can be found in any other region of the member, the leg is advanced with difficulty and the toe drops, we may conclude upon lameness of the shoulder, but the diagnosis is not positive.

The author recalls a case in which the lameness only manifested itself when the animal was pulling, and disappeared when led by the hand or trotted under the saddle. There was no alteration of the shoulder, and the lameness seemed to have been made apparent by the pressure of the collar.

The author divides the lameness of the shoulder into:

1. *Lameness without lesion.*

2. *Strain or Rupture of the Muscles.* In this case there is pain and lameness, but no local symptoms, such as swelling and pain on pressure.

3. *Shoulder-slip.* There is muscular atrophy, lameness, and a sudden outward rotation of the shoulder. As causes he mentions rupture of the fibres, of the superspinatic and subspinatic muscles, with atrophy and relaxation; also paralysis of the suprascapular nerve, which innervates these muscles. As a cause for the latter he suggests the pressure of the collar. Section of the tendon of the subspinatus muscle produces a similar rotation of the shoulder.

4. *Paralysis of the Olecranon Muscles.* This phenomenon may result from casting for operating ; also from a sudden effort in a hard pull, when the front feet slip and the animal forcibly tries to regain his feet, the lameness appearing almost instantaneously. The symptoms are dropping of the elbow and a flexed condition of the articulations below.

The lesions are : 1. Paralysis of the radial nerve. 2. Fracture of the first rib ; the latter condition has been revealed a number of times after necropsy ; it requires several weeks for the animal to recover.

5. *Arthritis and Bursitis.* The scapulohumeral articulation and the tendinous bursæ (biceps, subspinatus) may become inflamed. The symptoms are sometimes difficult of differentiation. The former (arthritis) is incurable, and the latter curable if the disease has not extended over six or eight weeks, by which time the cartilage on the trochanter has desquamated and the tendon ossified.

6. *Fractures.—L'Echo Vétérinaire.*

CYNANCHE PAROTIDÆ—MUMPS IN THE DOG. Cases of this disease have been mentioned by Whittaker, Hertwig, and Busquet (*Revue Vét.*, p. 663).

Busquet and Boudcaut have observed a recent case in a Bordeaux dog. *Incubation,* three to four days. *Symptoms :* Shivering, general weakness, inappetence, frequent chills, sneezing, snuffled breathing, cough, rapid tumefaction of the salivary glands, especially the parotid and submaxillary ; local pain and œdema of the skin ; stenosis duct hard, salient, and tumefied ; enlargement of the neighboring lymphatic glands ; buccal mucous membrane pale and dry ; saliva scant ; mastication of solid food painful. Entire course of the disease, twelve days.

The disease was transmitted to a ten-months-old fox-terrier dog, which had lived with the sick one and played with cotton tampons which were used to swab out its mouth.

A micrococcus under the form of a diplostreptococcus analogous to that found in mumps in man by Féré and Busquet in 1895 was found in the saliva and in the blood under the form of a diplococcus analogous to or identical with that found in man by Laveran and Catrin in 1893.—*Revue Vét.*

RAREFYING OSTEITIS AS A CAUSE OF FRACTURE. The observation is recorded by Jolly. The first phalanx was broken into four fragments without any appreciable cause. The principal alteration was situated close to the internal glenoid cavity whose cartilage was ulcerated.

Histological examination demonstrated that the entire bone was the seat of a rarefying osteitis, varying in intensity at different points, but was especially pronounced at the point of fracture. Joly and Vivien examined an apparently normal first phalanx. Centres of osteitis were found here and there, but this process was most marked in the compact articular layer at the upper end, and more so on the inside than on the outside.—*Annales de Méd. Vét.*

TRANSMISSION OF APHTHOUS FEVER TO THE HORSE. Monsarrat reports two such cases. The first one was a horse on a farm where cattle were affected with aphthous fever. On the inside of the upper lip was a large ulcerated surface, and around it about a dozen characteristic aphthous ulcerations smaller than in cattle. The contamination was effected in bridling the horse, the owner also taking care of his cattle, and did not take the precaution to wash his hands.

In the second case Monsarrat infected his own horse by touching the nostrils while inspecting a diseased herd. In three days inappetence, swelling of the nostrils, and aphthous ulcers upon superior lips and nasal mucous membrane were noticed. The recovery was rapid.—*Annales de Méd. Vét.*

PRIMARY ACTINOMYCOSIS OF THE BLADDER. The subject was an ox. The wall of the bladder was indurated, and on its peritoneal ecchymotic and bossellated. On the internal surface was an actinomycotic tumor as large as a fist, containing small, disseminated abscesses. The mucous membrane over the tumor was covered with numerous long, papillary vegetations, some of which were 3 to 4 cm. in length. Microscopical examination revealed the *actinomycosis bovis.* The probable mode of infection was through the urachus after birth, since the tumor occupied the fundus of the bladder and all the other organs were normal.—*Annales de Méd. Vét.*

ACUTE POLYARTHRITIS IN THE COW. According to Leblanc and Bitard arthritis is a frequent complication of parturition, abortion, and non-deliverance. It may develop under the acute or the chronic form. The femoro-tibial articulation is most frequently affected, but any of the other joints may become diseased.

Moussu, who has made a thorough anatomo-pathological study of this disease, recognizes two forms of the post-partum arthritis—an *exudative* and a *plastic*. In the former there is a hypersecretion of the synovial fluid ; in the latter the articular cavity contains little synovial fluid, but is filled with false membranes. The first form may sometimes change into the second. They can be differentiated from other forms of arthritis in that they are never purulent. Nine times out of ten the disease affected cows aborting the first or the second calf.

The channel of infection is generally accepted to be the uterus, but the microbe, which is the undoubted cause, has not yet been determined.—*Annales de Méd. Vét.*

ETIOLOGY OF EPIZOÖTIC OPHTHALMIA IN THE OX. On two farms Schmidt has seen the disease characterized essentially by a serous conjunctivitis and opacity of the cornea. After several days the centre of the cornea shows a suppurative point, and the conjunctiva secretes a purulent mucus. The centre of the cornea ulcerates, and is replaced by a grayish cicatrix. The eyeball otherwise generally remains normal. In some cases the anterior chamber of the eye becomes filled with a fibrinous, flocculent deposit, which covers the iris, although the latter is not the seat of inflammation. The symptoms are always benign and limited to one eye.

From the ulcerated cornea Schmidt has collected and cultivated in a pure state the staphylococcus pyogenes albus, and considered it the essential factor in the disease. The stables were infected, beside, with the microbes of pus ; in one of them a cow died from purulent infection after a prolonged decubitus from arthritis of the knee and stifle. A cow affected with a purulent wound of the knee had for a long time been in the other stable. The pyogenic germs were easily disseminated through the stable by the shaking up of the dusty straw.

The author made some inoculations of the pure cultures of staphylococcus in two rabbits and one calf with negative results.

After a complete disinfection of the stables there were no new cases of the disease.—*Revue Vétérinaire.*

---

THE McKillip Veterinary College of Chicago will have a valuable addition to its teaching faculty through Dr. John J. Miller, of Sioux City, who has connected himself with the college and will fill the chair of bovine medicine and obstetrics of the course of 1901–1902.

